# Factors associated with non-attendance, opportunistic attendance and reminded attendance to cervical screening in an organized screening program: a cross-sectional study of 12,058 Norwegian women

**DOI:** 10.1186/1471-2458-11-264

**Published:** 2011-04-26

**Authors:** Bo T Hansen, Silje S Hukkelberg, Tor Haldorsen, Tormod Eriksen, Gry B Skare, Mari Nygård

**Affiliations:** 1 Department of Screening-based Research, Cancer Registry of Norway, Oslo, Norway

## Abstract

**Background:**

Cervical cancer incidence and mortality may be reduced by organized screening. Participant compliance with the attendance recommendations of the screening program is necessary to achieve this. Knowledge about the predictors of compliance is needed in order to enhance screening attendance.

**Methods:**

The Norwegian Co-ordinated Cervical Cancer Screening Program (NCCSP) registers all cervix cytology diagnoses in Norway and individually reminds women who have no registered smear for the past three years to make an appointment for screening. In the present study, a questionnaire on lifestyle and health was administered to a random sample of Norwegian women. The response rate was 68%. To address the predictors of screening attendance for the 12,058 women aged 25-45 who were eligible for this study, individual questionnaire data was linked to the cytology registry of the NCCSP. We distinguished between non-attendees, opportunistic attendees and reminded attendees to screening for a period of four years. Predictors of non-attendance versus attendance and reminded versus opportunistic attendance were established by multivariate logistic regression.

**Results:**

Women who attended screening were more likely than non-attendees to report that they were aware of the recommended screening interval, a history of sexually transmitted infections and a history of hormonal contraceptive and condom use. Attendance was also positively associated with being married/cohabiting, being a non-smoker and giving birth. Women who attended after being reminded were more likely than opportunistic attendees to be aware of cervical cancer and the recommended screening interval, but less likely to report a history of sexually transmitted infections and hormonal contraceptive use. Moreover, the likelihood of reminded attendance increased with age. Educational level did not significantly affect the women's attendance status in the fully adjusted models.

**Conclusions:**

The likelihood of attendance in an organized screening program was higher among women who were aware of cervical screening, which suggests a potential for a higher attendance rate through improving the public knowledge of screening. Further, the lower awareness among opportunistic than reminded attendees suggests that physicians may inform their patients better when smears are taken at the physician's initiative.

## Background

Most cervical cancers are preceded by clinically asymptomatic preinvasive lesions that are caused by sexually transmitted human papillomaviruses (HPV) [1]. The mortality from cervical cancer has declined in several developed countries over the last decades [2], particularly in countries with organized cervical screening programs [3]. Cervical screening can reduce the cervical cancer incidence and mortality by detection and treatment of preinvasive lesions, and of invasive lesions at earlier, more curable stages.

The countries with the highest attendance to cervical screening have organized programs which keep track of the women's screening status and remind women to be screened. Most countries, however, do not have an organized screening program and thus rely on opportunistic screening in which the initiative to be screened is left to the women [2]. It has generally been found that the probability of not attending screening is higher among: older women [4], single women [5], women with low socio-economic status [6], women with little interaction with the health system [7] and women with little knowledge of screening [8]. Other factors that sometimes have been shown to be associated with screening attendance include ethnicity [9], psychological barriers [10], urban/rural residence and smoking [11].

Most of the studies that address attendance to cervical screening employ data from an opportunistic screening setting and rely on self-report of screening behavior [2]. Hence, there is a relative shortage of studies with quality-assured data on attendance, as well as studies that address the determinants of attendance in an organized screening setting. Even in countries with organized screening, many women are screened opportunistically, i.e. without receiving a reminder from the program, because they attend at least as frequently as the recommended interval. Little is known about what characterizes the different types of screening attendees in countries with an organized program.

The aim of the present study was to investigate factors associated with attendance and non-attendance to the organized cervical screening program in Norway. Such knowledge is necessary to increase attendance rates. We further wanted to investigate factors associated with reminded versus opportunistic attendance to screening in this setting.

## Methods

### Screening setting

The Norwegian Co-ordinated Cervical Cancer Screening Program (NCCSP) was initiated in 1995 and the Cancer Registry in Norway is responsible for the management of the program. All laboratories which analyze cytological smears in Norway are legally obliged to report the result of each smear to the NCCSP. Smear results are registered by a personal identification number (PIN) which is unique to each Norwegian citizen. The NCCSP individually reminds women aged 25-69 who have not had a smear for the past three years to make an appointment for smear-taking. Reminder letters also contain basic information about screening and cervical cancer. All women of eligible age are included in the NCCSP unless they have informed the program that they do not wish to participate, or have had a gynecological cancer diagnosis or have had their cervix removed. A woman receives her first letter from the NCCSP the year she turns 25. Eligible women who have no registered smear during the last three years receive a reminder 37 months after her last registered smear. If no smear is registered during the 12 months following the first reminder, a second reminder is sent 49 months after her last registered smear. The NCCSP does not contact women who have had a smear during the last three years. A woman has to pay circa € 20 for a smear at a general practitioner. There is no disincentive to more frequent screening than the NCCSP recommends.

### Data sources

A self-administrated questionnaire was mailed to a random sample of women aged 18-45 during November 2004 - June 2005 [12]. The questionnaire was also available on the web through the use of a personal access code. The survey was designed to establish baseline characteristics of lifestyle and health among females aged 18-45 before the introduction of the vaccine against HPV. It included women from Denmark, Iceland, Norway and Sweden, but the present study only included the Norwegian participants. The sample was randomly drawn by the National population register. A reminder was sent to women who did not respond within four weeks. Those who still did not respond were contacted by phone and asked to answer the questions in a telephone interview. The response rates were 66.2, 70.1, 69.3 and 67.0% for the age groups 25-29, 30-34, 35-39 and 40-45, respectively. Most women included in the study responded to the mailed questionnaire (80.6%), whereas 10.4% responded via the web-based questionnaire and 9.0% via interview.

All questions were pretested by an external group of women to ensure clarity. To gain information about screening attendance, the questionnaire data was linked by PIN to the NCCSP databases, which contain information about all smears taken in Norway.

### Survey participation

A total of 25,001 Norwegian women aged 18-45 years were asked to participate, among which 577 were excluded because they had died or were not reached at their registered address. The target population therefore consisted of 24,424 women. Among these, 2,409 women explicitly stated they did not want to participate in the study, and another 5,411 women did not return the questionnaire, leaving 16,604 responding women (68%). Individual information about the women not participating in the survey was limited to age and residence. Since women eligible for NCCSP are aged 25-69, responders below age 25 were excluded from this study (N = 4,428). In addition, 29 women with a discrepancy in the reported PIN, 20 women who had stated they did not want to receive reminders from the NCCSP, 49 women with gynecological cancer, and 20 women who had had their cervix removed were excluded.

The women were informed about the study in a cover letter which included statements that the data would be linked to health registries and that answering the questionnaire constituted consent to participate. Only authorized personnel at the Cancer Registry of Norway had access to the PINs of the women asked to participate in the survey. To protect the participants' privacy, all analyses were performed with de-identified data. The study was approved by the Norwegian Data Inspectorate and the Regional Committee for Medical and Health Research Ethics.

### Attendance definitions

Attendance to cervical screening was based on each woman's cytological smear history as recorded in the NCCSP databases. Women were categorized as attendees if they had had at least one smear during the last four years and as non-attendees if no smear was recorded in the same interval.

Opportunistic attendees were defined as women who were not sent a reminder by the NCCSP within one year prior to attendance, whereas reminded attendees were defined as women who were sent a first or second reminder within one year prior to attendance.

### Statistics

Odds ratios with corresponding 95% confidence intervals were derived from logistic regression models. The significance of independent variables was assessed by likelihood ratio tests, reported as P-values associated with a corresponding difference in deviance assuming a chi-square distribution.

The α-level was 0.05. Non-attendance versus attendance, and reminded attendance versus opportunistic attendance were modeled as separate dichotomous response variables. For each response variable, we performed one age-adjusted model per independent variable, as well as one fully adjusted model in which all independent variables that proved significant in the age-adjusted models were included.

## Results

Overall, non-participants and participants in the survey were similar with respect to age, residential region and the median household income of their residential municipality, although a slightly higher proportion of non-participants than participants lived in Oslo and in municipalities with the lowest median income (Table 1). A total of 12,058 women participated in the survey. Their mean age was 34.8 years (SD = 5.9). A total of 13.4% (N = 1,614) of the participating women had not attended screening during the last four years, and were hence classified as non-attendees (Table 2). The remaining 86.6% (N = 10,444) had attended screening during the last four years and were classified as attendees. Among attendees, 51.5% (N = 5,375) were opportunistic attendees and 48.5% (N = 5,069) were reminded attendees. Overall, there was good concordance in attendance status between the study participants and the total population in 2004, although the rate of women classified as non-attendees was somewhat higher in the total population (Table 2).

**Table 1:** Characteristics of non-participants and participants in the questionnaire survey

	Frequency (%) among non- participants (N = 5,628)	Frequency (%) among participants (N = 12,058)
**Age group (years)**		
25-29	25.3	23.4
30-34	22.7	24.7
35-39	24.1	25.3
40-45	27.9	26.7
**Residential region in Norway**		
Oslo (capital)	18.1	15.4
Eastern (minus Oslo)	36.9	38.5
Western	29.2	28.7
Northern	15.8	17.4
**Median income in residential municipality (NOK)^1^**		
240,000-290,000	20.2	17.8
290,001-310,000	18.4	17.7
310,001-320,000	17.3	17.1
320,001-360,000	22.1	23.0
360,001-450,000	22.0	24.3

^1 ^Data from Statistics Norway on the 2005 median income per household in the women's residential municipality. There are 430 municipalities in Norway

**Table 2:** Attendance status according to age (N, %)

Age	Nonattendees		Spontaneous attendees		Reminded attendees		Total	
	**Study sample**	**Total population** **2004**	**Study sample**	**Total population** **2004**	**Study sample**	**Total population** **2004**	**Study sample**	**Total population** **2004**

25 - 29	502 (17.8)	44,885 (28.2)	1,406 (49.9)	63,830 (40.1)	912 (32.3)	50,592 (31.7)	2,820 (100)	159,307 (100)
30 - 34	347 (11.7)	36,407 (20.4)	1,401 (47.0)	74,559 (41.8)	1,230 (41.3)	67,270 (37.8)	2,978 (100)	178,236 (100)
35 - 39	375 (12.3)	35,684 (20.1)	1,275 (41.9)	68,215 (38.4)	1,395 (45.8)	73,792 (41.5)	3,045 (100)	177,691 (100)
40 - 45	390 (12.1)	36,900 (20.7)	1,293 (40.2)	63,710 (35.8)	1,532 (47.7)	77,554 (43.5)	3,215 (100)	178,164 (100)
Total	1,614 (13.4)	153,876 (22.2)	5,375 (44.6)	270,314 (39.0)	5,069 (42.0)	269,208 (38.8)	12,058 (100)	693,398 (100)

### Non-attendance versus attendance

Age influenced the level of attendance to cervical screening in a univariate model, mainly through a lower attendance in the youngest age group than in the older age groups. However, this effect disappeared in the fully adjusted model (Table 3). The level of attendance decreased with a decreasing level of education. This association proved significant in the age-adjusted model, but not in the fully adjusted model (Table 3). Women with < 9, 9-12 and 13-16 years of education did not differ in attendance in the latter model, but women with >16 years of education had a somewhat higher level of attendance than women with 13-16 years of education. Marital status strongly affected attendance in the age-adjusted model, in which divorced/widowed women and single women were less likely to attend than married/cohabiting women (Table 3). Marital status similarly influenced attendance in the fully adjusted model, but here only the single women were significantly less likely to attend than the married/cohabiting women.

**Table 3:** Odds ratios (OR) and 95% confidence intervals (CI) for non-attendance versus attendance to cervical screening

	N	Non-attendance (%)	Age-adjusted OR (95% CI) for non-attendance	**Multivariate**^**a **^**OR** (95% CI) for non-attendance
**SOCIODEMOGRAPHICS**				
**Age**				
25-29	2820	17.80	1	1
30-34	2978	11.65	0.61 (0.53, 0.71)	0.92 (0.76, 1.12)
35-39	3045	12.32	0.65 (0.56, 0.75)	1.14 (0.93, 1.40)
40+	3215	12.13	0.64 (0.55, 0.74)	1.00 (0.80, 1.25)
*P-value*			*P < 0.0001*	*P = 0.21*
**Education (years)**				
< 9	258	20.54	1.79 (1.29, 2.43)	0.94 (0.57, 1.48)
9-12	3226	15.22	1.26 (1.11, 1.43)	1.04 (0.88, 1.23)
13-16	5235	12.91	1	1
>16	3238	11.83	0.91 (0.79, 1.04)	0.81 (0.69, 0.96)
*P-value*			*P < 0.0001*	*P = 0.06*
**Marital status**				
Married/cohabiting	9201	11.27	1	1
Divorced/widow	784	14.54	1.40 (1.13, 1.73)	1.15 (0.87, 1.52)
Single	1975	22.78	2.18 (1.92, 2.47)	1.38 (1.16, 1.65)
*P-value*			*P < 0.0001*	*P = 0.002*
**HEALTH AND HEALTH RISKS**				
**Self-rated health**				
Poor	852	19.01	1.84 (1.51, 2.22)	1.26 (0.97, 1.63)
Good	3645	14.32	1.28 (1.13, 1.45)	1.16 (0.99, 1.35)
Very good	5494	11.65	1	1
Excellent	1809	14.26	1.27 (1.09, 1.49)	1.38 (1.14, 1.66)
*P-value*			*P < 0.0001*	*P = 0.005*
**Smoking**				
Never	5435	13.21	1	1
Former	2684	9.84	0.73 (0.63, 0.85)	0.94 (0.78, 1.13)
Current	3899	16.03	1.26 (1.12, 1.42)	1.41 (1.20, 1.66)
*P-value*			*P < 0.0001*	*P < 0.0001*
**Beer drinking**				
Never	2626	11.00	0.96 (0.82, 1.12)	
<once per month	3336	13.01	1.08 (0.93, 1.24)	
1-3 times per month	3788	12.46	1	
>=once per week	944	13.45	1.08 (0.87, 1.33)	
*P-value*			*P = 0.46*	
**Wine drinking**				
Never	966	17.39	1.55 (1.28, 1.87)	1.34 (1.06, 1.68)
<once per month	2884	14.08	1.22 (1.06, 1.40)	1.13 (0.96, 1.33)
1-3 times per month	4947	11.91	1	1
>=once per week	2187	9.83	0.84 (0.71, 0.99)	0.93 (0.77, 1.12)
*P-value*			*P < 0.0001*	*P = 0.03*
**Liquor drinking**				
Never	3597	12.29	1.02 (0.89, 1.17)	
<once per month	4716	12.30	1	
>=once per month	1827	12.97	1.03 (0.87, 1.21)	
*P-value*			*P = 0.92*	
**SEXUAL HISTORY**				
**Number of lifetime coital partners**				
0-3^b^	3992	15.98	1	1
4-9	4265	12.64	0.76 (0.67, 0.86)	1.02 (0.87, 1.21)
10 +	3423	11.01	0.65 (0.57, 0.74)	0.85 (0.69, 1.04)
*P-value*			*P < 0.0001*	*P = 0.09*
**Number of new coital partners during last six months**				
0	9696	13.26	1	
1	1691	14.02	1.03 (0.89, 1.20)	
2+	572	12.94	0.90 (0.69, 1.16)	
*P-value*			*P = 0.65*	
**Age at first coitus**				
<17	4997	11.43	1	1
17-18	4023	13.05	1.18 (1.04, 1.34)	1.07 (0.92, 1.26)
19 +	2673	14.18	1.28 (1.12, 1.47)	0.99 (0.81, 1.20)
*P-value*			*P = 0.001*	*P = 0.54*
**Ever had any STI^c^**				
No	8061	15.15	1	1
Yes	3627	9.15	0.57 (0.50, 0.64)	0.66 (0.56, 0.78)
*P-value*			*P < 0.0001*	*P < 0.0001*
**PREGNANCY AND CONTRACEPTIVES**				
**Ever pregnant**				
No	2300	23.57	2.44 (2.14, 2.76)	1.21 (0.94, 1.56)
Yes	9720	10.92	1	1
*P-value*			*P < 0.0001*	*P = 0.13*
**Number of births**				
0	3133	21.54	1	1
1	2307	11.10	0.46 (0.39, 0.54)	0.62 (0.48, 0.82)
2	4013	9.54	0.38 (0.33, 0.44)	0.57 (0.44, 0.74)
3+	2419	10.87	0.44 (0.37, 0.52)	0.59 (0.44, 0.78)
*P-value*			*P < 0.0001*	*P = 0.0006*
**Ever used hormonal contraceptives**				
No	1305	31.72	3.84 (3.36, 4.38)	2.12 (1.75, 2.58)
Yes	10670	11.12	1	1
*P-value*			*P < 0.0001*	*P < 0.0001*
**Used hormonal contraceptive during last month**				
No	7825	15.49	1	1
Yes	4097	9.37	0.53 (0.47, 0.60)	0.69 (0.60, 0.81)
*P-value*			*P < 0.0001*	*P < 0.0001*
**Ever used condoms**				
No	1873	21.41	2.13 (1.87, 2.42)	1.38 (1.15, 1.65)
Yes	10079	11.81	1	1
*P-value*			*P < 0.0001*	*P = 0.0007*
**AWARENESS**				
**"Did you know that a gynecological smear may detect changes that could lead to cervical cancer?"**				
No	1224	23.37	2.04 (1.76, 2.35)	1.20 (0.96, 1.50)
Yes	10769	12.20	1	1
*P-value*			*P < 0.0001*	*P = 0.11*
**"Did you know that the recommended screening interval is every third year?"**				
No	1179	25.36	2.31 (1.99, 2.67)	1.39 (1.12, 1.72)
Yes	10835	12.04	1	1
*P-value*			*P < 0.0001*	*P = 0.003*
**"Do you believe a gynecological smear every third year is necessary for you?"**				
No	8733	9.58	0.50 (0.44, 0.56)	0.52 (0.45, 0.61)
Yes	2382	17.72	1	1
Don't know	883	38.05	2.75 (2.32, 3.27)	2.19 (1.75, 2.74)
*P-value*			*P < 0.0001*	*P < 0.0001*
**"Have you ever heard of HPV?"**				
No	7664	14.08	1.22 (1.09, 1.36)	0.98 (0.85, 1.13)
Yes	4290	11.91	1	1
*P-value*			*P = 0.0006*	*P = 0.73*

^a ^The multivariate model only included variables that were significant in the age-adjusted models

^b ^Included 154 women reporting zero lifetime coital partners

^c ^Chlamydia, gonorrhea, trichomonas vaginalis, herpes or genital warts

Self-rated health was associated with attendance in the age-adjusted model, in which women reporting poor, good or excellent health all attended less than women reporting very good health (Table 3). The same pattern was evident in the fully adjusted model, although the individual contrasts in some cases fell short of significance. Smoking influenced attendance in both kinds of models (Table 3). In the age-adjusted model, former smokers attended more and current smokers attended less than never smokers. In the fully adjusted model, former smokers did not differ from never smokers, but current smokers attended significantly less than never smokers. The frequency of beer and liquor drinking did not differ between attendees and non-attendees (Table 3). Wine drinking habits, on the other hand, were associated with attendance (Table 3). In the age-adjusted model, the likelihood to attend increased with an increasing frequency of wine drinking. Wine drinking significantly influenced attendance in the fully adjusted model too, although the only significant single contrast was the lower attendance among never-drinkers compared to women drinking wine 1-3 times per month.

There was an increase in attendance with an increasing number of lifetime coital partners, but this relationship did not reach significance in the fully adjusted model (Table 3). The number of recently acquired coital partners was not associated with attendance (Table 3). Increasing age at coital debut was associated with a decrease in attendance in the age-adjusted model, but not in the fully adjusted model (Table 3). Ever having been diagnosed with an STI was strongly associated with attendance both in the age-adjusted and the fully adjusted model; women who had been diagnosed with an STI were more likely to have attended screening than were women who had not been diagnosed with an STI (Table 3).

Women who ever had been pregnant had a somewhat higher level of attendance than women who never had been pregnant, but this association proved significant only in the age-adjusted model (Table 3). Women who had given birth were more likely to have attended screening than women who had not given birth, both in the age-adjusted and the fully adjusted model, although there did not seem to be an increase in attendance with an increasing number of births (Table 3). Ever having used hormonal contraceptives was among the strongest predictors of attendance, ever-use being associated with a far higher attendance rate than never-use in both kinds of models (Table 3). Similarly, recent hormonal contraceptive use and ever-use of condoms were associated with higher attendance (Table 3).

Women who knew that a gynecological smear may detect changes that could lead to cervical cancer had a higher attendance rate than women without this knowledge, but the association was significant only in the age-adjusted model (Table 3). Having knowledge of the recommended screening interval was associated with higher attendance, even in the fully adjusted model (Table 3).

The strongest predictor of cervical screening attendance in our data was the women's opinion on the necessity to have a gynecological smear every third year (Table 3). Women who did not believe that a test every third year was necessary for them attended screening more than women who believed it was necessary. In contrast, women who answered they did not know attended screening less frequently than women who believed it was necessary. These effects were pronounced and highly significant even in the fully adjusted model. Finally, having heard of HPV was associated with a somewhat higher attendance, but this association did not reach significance in the fully adjusted model (Table 3).

### Reminded attendance versus opportunistic attendance

Whether women attended screening opportunistically or after receiving a reminder from the screening program depended on age, since the level of reminded attendance generally increased with age, both in the age-adjusted and in the fully adjusted model (Table 4). However, we did not find any effect of the women's educational level (Table 4). Divorced/widowed as well as single women tended to have a lower level of reminded attendance than married/cohabiting women, although this association was not significant in the fully adjusted model (Table 4). Self-rated health, smoking and alcohol drinking did not influence the level of reminded versus opportunistic attendance in the fully adjusted model (Table 4).

**Table 4:** Odds ratios (OR) and 95% confidence intervals (CI) for attendance to cervical screening after receipt of a reminder, versus opportunistic attendance on own initiative

	N	Reminded attendance (%)	Age-adjusted OR (95% CI) for reminded attendance	Multivariate^a ^OR (95% CI) for reminded attendance
**SOCIODEMOGRAPHICS**				
**Age**				
25-29	2318	39.34	1	1
30-34	2631	46.75	1.35 (1.21, 1.52)	1.24 (1.09, 1.41)
35-39	2670	52.25	1.69 (1.51, 1.89)	1.58 (1.39, 1.80)
40+	2825	54.23	1.83 (1.63, 2.04)	1.66 (1.45, 1.90)
*P-value*			*P < 0.0001*	*P < 0.0001*
**Education (years)**				
< 9	205	49.76	1.06 (0.80, 1.40)	
9-12	2735	50.16	1.02 (0.92, 1.12)	
13-16	4559	48.41	1	
>16	2885	47.25	1.02 (0.88, 1.06)	
*P-value*			*P = 0.77*	
**Marital status**				
Married/cohabiting	8164	49.47	1	1
Divorced/widow	670	47.31	0.82 (0.70, 0.96)	0.88 (0.73, 1.06)
Single	1525	44.59	0.90 (0.81, 1.01)	0.95 (0.83, 1.08)
*P-value*			*P = 0.01*	*P = 0.33*
**HEALTH AND HEALTH RISKS**				
**Self-rated health**				
Poor	690	46.23	0.86 (0.73, 1.00)	
Good	3123	47.26	0.93 (0.85, 1.01)	
Very good	4854	49.01	1	
Excellent	1551	49.39	1.00 (0.89, 1.12)	
*P-value*			*P = 0.12*	
**Smoking**				
Never	4717	50.03	1	1
Former	2420	47.07	0.86 (0.78, 0.95)	0.97 (0.87, 1.09)
Current	3274	47.37	0.88 (0.81, 0.97)	1.03 (0.92, 1.15)
*P-value*			*P = 0.003*	*P = 0.66*
**Beer drinking**				
Never	2311	49.94	1.16 (1.04, 1.29)	1.05 (0.93, 1.18)
<once per month	2902	49.93	1.13 (1.02, 1.25)	1.05 (0.94, 1.17)
1-3 times per month	3316	46.47	1	1
>=once per week	817	45.53	0.96 (0.82, 1.12)	1.03 (0.87, 1.22)
*P-value*			*P = 0.008*	*P = 0.84*
**Wine drinking**				
Never	798	50.88	1.19 (1.02, 1.38)	1.17 (0.98, 1.39)
<once per month	2478	50.12	1.13 (1.02, 1.24)	1.08 (0.96, 1.21)
1-3 times per month	4358	47.22	1	1
>=once per week	1972	47.36	0.92 (0.83, 1.03)	0.96 (0.85, 1.08)
*P-value*			*P = 0.002*	*P = 0.13*
**Liquor drinking**				
Never	3155	49.57	1.01 (0.92, 1.11)	
<once per month	4136	48.77	1	
>=once per month	1590	46.04	0.92 (0.82, 1.03)	
*P-value*			*P = 0.26*	
**SEXUAL HISTORY**				
**Number of lifetime coital partners**				
0-3^b^	3354	54.03	1	1
4-9	3726	47.07	0.76 (0.70, 0.84)	0.88 (0.79, 0.99)
10 +	3046	44.42	0.69 (0.62, 0.76)	0.87 (0.76, 0.99)
*P-value*			*P < 0.0001*	*P = 0.05*
**Number of new coital partners during last six months**				
0	8410	49.27	1	
1	1454	46.77	0.94 (0.84, 1.05)	
2+	498	42.77	0.84 (0.70, 1.01)	
*P-value*			*P = 0.11*	
**Age at first coitus**				
<17	4426	46.07	1	1
17-18	3498	48.66	1.10 (1.01 1.20)	1.07 (0.97, 1.19)
19 +	2294	52.14	1.26 (1.14, 1.40)	1.07 (0.94, 1.22)
*P-value*			*P < 0.0001*	*P = 0.36*
**Ever had any STI^c^**				
No	6840	51.40	1	1
Yes	3295	42.12	0.68 (0.63, 0.74)	0.76 (0.68, 0.83)
*P-value*			*P < 0.0001*	*P < 0.0001*
**PREGNANCY AND CONTRACEPTIVES**				
**Ever pregnant**				
No	1758	45.68	1.08 (0.97, 1.21)	
Yes	8659	49.16	1	
*P-value*			*P = 0.17*	
**Number of births**				
0	2458	45.04	1	
1	2051	46.03	0.92 (0.82, 1.04)	
2	3630	49.89	0.96 (0.86, 1.08)	
3+	2156	52.97	1.03 (0.90, 1.17)	
*P-value*			*P = 0.35*	
**Ever used hormonal contraceptives**				
No	891	57.35	1.39 (1.21, 1.60)	1.21 (1.01, 1.46)
Yes	9483	47.63	1	1
*P-value*			*P < 0.0001*	*P = 0.03*
**Used hormonal contraceptive during last month**				
No	6613	50.20	1	1
Yes	3713	45.33	0.87 (0.80, 0.95)	0.88 (0.80, 0.96)
*P-value*			*P = 0.001*	*P = 0.006*
**Ever used condoms**				
No	1472	54.28	1.23 (1.10, 1.38)	1.07 (0.94, 1.23)
Yes	8889	47.54	1	1
*P-value*			*P = 0.0003*	*P = 0.31*
**AWARENESS**				
**"Did you know that a gynecological smear may detect changes that could lead to cervical cancer?"**				
No	938	40.94	0.78 (0.68, 0.90)	0.78 (0.65, 0.94)
Yes	9455	49.25	1	1
*P-value*			*P = 0.0006*	*P = 0.007*
**"Did you know that the recommended screening interval is every third year?"**				
No	880	33.18	0.54 (0.47, 0.63)	0.49 (0.41, 0.59)
Yes	9531	49.96	1	1
*P-value*			*P < 0.0001*	*P < 0.0001*
**"Do you believe a gynecological smear every third year is necessary for you?"**				
No	7896	49.23	1.14 (1.03, 1.26)	1.06 (0.93, 1.20)
Yes	1960	45.66	1	1
Don't know	547	49.73	1.25 (1.04, 1.52)	1.13 (0.89, 1.43)
*P-value*			*P = 0.02*	*P = 0.54*
**"Have you ever heard of HPV?"**				
No	6585	49.70	1.14 (1.05, 1.24)	1.19 (1.08, 1.30)
Yes	3779	46.44	1	1
*P-value*			*P = 0.001*	*P = 0.0003*

^a ^The multivariate model only included variables that were significant in the age-adjusted models

^b ^Included 154 women reporting zero lifetime coital partners

^c ^Chlamydia, gonorrhea, trichomonas vaginalis, herpes or genital warts

Women who reported 0-3 lifetime coital partners had a higher level of reminded attendance than women who reported more partners, both in the age-adjusted and the fully adjusted models (Table 4). There were no significant associations for the number of recently acquired partners (Table 4). An increasingly advanced age at first coitus was associated with a higher level of reminded attendance, but significantly so only in the age-adjusted model (Table 4). One of the strongest predictors of reminded versus opportunistic attendance was ever having been diagnosed with an STI (Table 4). Women who reported a previous STI diagnosis had a lower level of reminded attendance than did women who reported not to have been diagnosed with an STI.

Ever having been pregnant did not influence the level of reminded versus opportunistic screening attendance (Table 4). The same was true for the number of births given (Table 4). Women who reported to ever have used hormonal contraceptives had a lower level of reminded attendance than women who never had used hormonal contraceptives (Table 4). Similarly, women who had used hormonal contraceptives during the last month were less likely to have been reminded to attend than were women who had not used it recently (Table 4). Both predictors of hormonal contraceptive use proved significant in the age-adjusted as well as the fully adjusted model. Ever-use of condoms was associated with a lower level of reminded attendance compared to never-use, but significantly so only in the age-adjusted model (Table 4).

Women with knowledge that a smear may detect changes that could lead to cancer and women with knowledge about the recommended screening interval were more likely to have received a reminder before they attended screening than women lacking this knowledge, both in the age-adjusted and in the fully adjusted model (Table 4). However, the women's opinion on the necessity for them to have a smear every third year did not significantly influence the fully adjusted model (Table 4). Finally, women who had not heard of HPV were more likely to have received a reminder before attending screening than were women who had heard of HPV (Table 4).

## Discussion

### Awareness of screening and cervical cancer

More than 90% of the women were familiar with the function of the cervical smear and the recommended screening interval. Having this knowledge markedly increased attendance. However, the effects were weakened in the fully adjusted model on non-attendance versus attendance, showing that knowledge of screening was associated with some of the other variables that also were associated with the outcome. The same variables were also strong predictors of reminded versus opportunistic attendance, and attending women who were unaware of the smear and of the recommended screening interval were more likely to have attended opportunistically. Thus, some opportunistically screened women probably had a smear at the initiative of their physician without appreciating the rationale for the test. This suggests a need for more screening information from the physician, especially when the smear is taken at the physician's initiative. Further, our finding indicates that reminders containing basic facts about screening may have an educational function.

The answer to the question "Do you believe a gynecological smear every third year is necessary for you?" was the strongest predictor of non-attendance versus attendance. Nearly two thirds of the women answered negatively to the question and, surprisingly, they were more likely to attend than women who answered positively. This finding indicates that the motivation for attendance often does not depend strongly on the perception of being at risk for cervical cancer. A negative answer may reflect awareness that most women are not treated for cervical cancer or its precursors, a fact that often will be supported by personal experience. Women who regularly have attended screening and have had normal smears may perceive that they are not at high risk, and consequently that the recommended screening interval in their case is too short. Further, the high frequency of negative answers suggests that most women readily will accept an increased screening interval, which is a likely scenario if HPV-testing is introduced in primary screening [13]. The fact that many non-attendees believed that attendance at the recommended interval was necessary for them suggests that they have motivation to be screened, which is encouraging in terms of the efforts to increase attendance. However, women who did not attend were also far more likely not to have an opinion on the subject, which probably reflects low awareness of cervical screening.

### Risk behavior

It is important to know whether screening attendance differs according to established risk factors for cervical cancer. We found that current smokers were more likely to be non-attendees. Since smoking seems to be an independent co-factor for HPV progression [14], this indicates that non-attendees to cervical screening may be at an additional risk because they are more likely to be current smokers.

Sexual behavior is a major risk factor for HPV infection [1] and thus the development of cervical cancer, but we found no indication that women who reported a high number of partners, or who had their coital debut at an early age, had a lower attendance rate than other women. Other studies have also failed to find such associations [11,15]. However, women who reported never to have used condoms attended screening less than women who had used condoms. Condoms may offer some protection against the development of HPV-related lesions [16] and it is thus possible that non-attendees to cervical screening in Norway may be at a higher risk for HPV exposure through less condom use. Never-use of condoms may, however, be associated with having had few sex partners, but note that the association to non-attendance was significant when controlling for other variables, including the number of partners. It is thus possible that never-use of condoms in our data is indicative of a low level of health consciousness.

Women who ever had an STI and women who had used hormonal contraceptives were more likely to have attended screening, and they were also more likely to have attended screening opportunistically. Being diagnosed with an STI requires a gynecological examination, and purchase of hormonal contraceptives requires a prescription, thus both predictors are associated with seeing a physician. This may have influenced screening behavior in two ways. First, a smear may have been taken as part of the consultation regarding STI or hormonal contraceptives. This may also be an explanation for why women who have given birth were more likely to attend [17]. Second, women who use hormonal contraceptives, or see a physician to be tested for STIs (some of which are largely asymptomatic), may be more health conscious than women who do not and may hence be more inclined to seek preventive healthcare.

### Sociodemographic variables

Socio-economic status, as defined by social class, education or income level, has consistently been shown to be inversely related to the risk of cervical cancer [18-20]. Women of low socio-economic status also seem to have a lower attendance to cervical screening [2,21,22]. Since one of the motivations for organizing a screening program is to decrease social inequalities in the use of preventive health services it is possible that the socio-economic status differential is not equally relevant in organized and opportunistic screening settings. Interestingly, Eaker et al. [11] did not find an effect of socio-economic status on attendance to screening in a Swedish county with an organized screening program, and a recent study from Great Britain indicates that self-reported ever-attendance to organized cervical screening may not depend strongly on a number of socio-economic status variables, but is clearly affected by ethnicity [23]. In a similar vein, we did not find a significant effect of education in our fully adjusted model of non-attendance versus attendance. However, we cannot conclude that socio-economic status plays no role in attendance to cervical screening in an organized screening setting with a relatively high attendance rate. Firstly, our raw data and our age-adjusted analysis indicated that the attendance rate was increasing with the level of education. Secondly, educational level just fell short of significance in the fully adjusted model of non-attendance, and the contrast between the highest level of education and the reference level for the term was in fact significant even in this model. Thirdly, we only addressed educational level, which, although commonly used, does not entirely capture socio-economic status. Thus, we may conclude that in Norway, the educational level of women is not strongly associated with non-attendance to cervical screening when controlling for other factors. This issue needs to be further investigated, and it would be of interest to also include information on income, social class and ethnicity in such an analysis.

Consistent with previous reports [5,24], we found that single women were less likely to attend screening. Single women may have experienced less encouragement from a partner to seek preventive healthcare, and may also have received less obstetric care. Like in most developed countries [25], women in the youngest age group (25-29 years old) had a lower crude attendance rate than older women. However, any effect of age disappeared in the fully adjusted model of non-attendance versus attendance, demonstrating that some other variables, also associated with age, explained the lower attendance of the youngest women. Many of the key variables that influenced attendance in our models were also associated with age, such as marital status and history of giving birth.

Women increasingly relied on a reminder with increasing age. This could result from experience with the screening program. For instance, awareness that reminders will be sent may result in an increased reliance on reminders. Relatively older women may also perceive themselves to be at a low risk of sexually transmitted cervical disease and thus delay their screening visits, or be more prone to underestimate the time since they had their last smear.

### Strengths and limitations

The representativeness of our study sample is enhanced by the relatively high response rate and large sample size. Moreover, the women invited to participate in the survey were randomly drawn from the whole of Norway, thus our results should be generalizable to the Norwegian setting, and perhaps also to other countries with a similar organized screening program. A further strength is the quality-assured data on attendance. Many previous studies on attendance to cervical screening rely on self-report of screening behavior [4,26], which is imprecise [27,28]. The complete registration of screening attendance further allowed us to separately address opportunistic versus reminded attendance in an organized screening setting and we are not aware that such data has been published previously.

The study also has a number of limitations. First, the attendance rate in the study sample was somewhat higher than in the NCCSP. This is a universal problem in population-based studies of attendance, probably because those participating in a survey also are more inclined to participate in screening. However, the discrepancy was relatively small, presumably due to the high response rate in this study. We have limited information about the women who did not participate in the survey, but they seemed similar to participants with respect to age, region of residence and median household income in the municipality in which they lived. Another limitation is that our study sample was limited to 25-45 year old women. The screening program in Norway targets women up to the age of 69 and future studies should address predictors of attendance among women above age 45. Moreover, the study is cross-sectional, which is suboptimal to address causality, and the data on the women's lifestyle was based on self-report which may be prone to social desirability bias. Finally, we did not have data on the ethnicity of respondents, which may be of importance for attendance [23].

## Conclusions

We show that attendance to cervical screening in an organized screening program was positively associated with awareness of screening, having had an STI, use of hormonal contraceptives, use of condom, giving birth, being a non-smoker and being married/cohabiting. Since women with low awareness of screening were more likely to be non-attendees, our results indicate that increasing the awareness of cervical screening in the population may increase the attendance rate. The likelihood of being a reminded rather than an opportunistic attendee was positively associated with age, awareness of screening and cervical cancer, not having used hormonal contraceptives and not having had an STI. The lower awareness among opportunistic than reminded attendees suggests that physicians may inform their patients better when smears are taken at the physician's initiative.

## Competing interests

The authors declare that they have no competing interests.

## Authors' contributions

MN contributed to designing the questionnaire, GBS and MN coordinated the data collection, TE linked the questionnaire data to the NCCSP databases. SSH, TH, MN and BTH designed the study. BTH did the statistical analyses and wrote the manuscript. All authors revised the manuscript and read and approved the final version of it.

## Pre-publication history

The pre-publication history for this paper can be accessed here:

http://www.biomedcentral.com/1471-2458/11/264/prepub
